# Role of intraoperative cholangiography for detecting residual stones after biliary pancreatitis: still useful? A retrospective study

**DOI:** 10.1186/s13017-017-0130-9

**Published:** 2017-04-20

**Authors:** Abdelrahman Abdelaal, Moamena El-Matbouly, Ibnouf Sulieman, Ahmad Elfaki, Tamer El-Bakary, Sherif Abdelaziem, Salahdin Gehani, Adriana Toro, Isidoro Di Carlo

**Affiliations:** 1 Department of General Surgery, Hamad General Hospital, Al Rayyan Road, 3050 Doha, Qatar; 2 Department of Surgery, Barone Romeo Hospital, via Mazzini 14, 98066 Patti, (ME) Italy; 3 Department of Surgical Sciences and Advanced Technologies “G.F. Ingrassia”, University of Catania, Via Santa Sofia 78, 95100 Catania, Italy

**Keywords:** Acute biliary pancreatitis, Intraoperative cholangiography, Common bile duct, Laparoscopic cholecystectomy, Intraoperative common bile duct exploration

## Abstract

**Background:**

Intraoperative cholangiography (IOC) may detect residual stones in the common bile duct (CBD) after acute biliary pancreatitis (ABP). The aim of the present study is to analyze the utility of IOC in detecting residual stones in patients undergoing cholecystectomy for ABP and if complications are related with this procedure.

**Methods:**

Demographic and clinical factors were assessed in patients with mild ABP who underwent IOC during laparoscopic cholecystectomy. Factors assessed included preoperative size of the CBD on ultrasonography, presence of stones in the gallbladder and the CBD, and IOC results. For the statistical analysis, *χ* ^2^ or Fisher’s exact tests to compare proportions and the nonparametric Mann–Whitney *U* test for analysis of values with abnormal distribution were used.

**Results:**

The study included 113 patients, 82 males (72.6%) and 31 females (27.4%), of mean age 46.9 **±** 14.7 years (range 18–86 years). All preoperative laboratory indicators were elevated. The group of the patients with stones in the CBD diagnosed by IOC was divided in patients with diameters <0.8 mm and with diameters ≥0.8 mm of the CBD diagnosed preoperatively with ultrasound. The laboratory tests do not demonstrate difference statistically significative between these two groups. The group of the patients without stones in the CBD diagnosed by IOC was also divided in patients with diameters <0.8 mm and with diameters ≥0.8 mm of the CBD. Also in these two groups, the statistical analysis of the laboratory tests does not demonstrate significative difference. Most procedures were performed by specialists (64.6%), and all patients underwent IOC. IOC showed stones in 84/113 patients (74.3%). A comparison of patients with and without stones at IOC showed similar mean times from hospitalization to surgery (5.9 days [range 2–12 days] vs. 6.1 days [range 2–23 days]), from surgery until hospital discharge (2.0 days [range 0–4 days] vs. 2.2 days [range 0–11 days]), and overall length of stay (7.9 days [range 3–19 days] vs. 8.3 days [range 3–23 days]) (*P* > 0.001).

**Conclusions:**

IOC is useful to diagnose residual CBD stones, without increasing complications related to the procedure itself.

## Background

One of the consequences of gallbladder stones is acute biliary pancreatitis (ABP), present worldwide in 40% of patients diagnosed with pancreatitis [1]. Mild ABP, defined when 2 of the 3 criteria (clinical, laboratory, or imaging) are present, is caused by passage of the stones from the gallbladder to the common bile duct (CBD) [2], by obstruction of the ampulla of Vater, and by the reflux of bile in the pancreatic duct [1]. Most stones are small and pass spontaneously without further clinical consequences. However, stones that persist in the CBD can result in severe complications

Laparoscopic cholecystectomy (LC) is the technique of choice for removing gallbladder stones, but its timing is unclear. Performing LC 6 to 8 weeks after the onset of ABP may reduce both acute inflammation and the conversion rate [3]. In contrast, performing LC 48 h after hospital admission may reduce morbidity rates and hospital expenses [4].

As stones may persist in the CBD after ABP, intraoperative cholangiography (IOC) may be performed during LC to detect these stones [5]. This may allow their removal by CBD exploration or by intra- or postoperative endoscopic retrograde cholangiopancreatography (ERCP) [2]. In many studies, however, IOC has failed to detect stones, suggesting that IOC may not be useful in these patients. Thus, there is little evidence that IOC may be useful to diagnosis residual CBD stones, despite the guidelines recommending the routine performance of IOC in patients undergoing LC after ABP [2]. The aim of the present study is to analyze the utility of IOC in detecting residual stones in patients undergoing cholecystectomy for ABP and if complications are related with this procedure.

## Methods

This retrospective study included patients with mild ABP who underwent LC and IOC for residual stones, at the Department of Surgery of Hamad General Hospital, Doha, Qatar, over a 5-year period from 2010 to 2015. The demographic and clinical characteristics of these patients, including age, sex, nationality, abdominal pain, jaundice, fever, and chills before or during admission were recorded. Laboratory tests include preoperative serum concentrations of total bilirubin, alkaline phosphatase (ALP), aspartate aminotransferase (AST), alanine aminotransferase (ALT), gamma glutamyltransferase (GGT), amylase, and lipase were recorded. Only for laboratory tests, median and 25°–75° percentiles were considered for statistical analysis.

Other factors analyzed at preoperative ultrasonography were CBD size (diameters >0.8 mm was considered as dilated) and the presence of gallbladder stones and/or stones in the CBD.

Operative factors included the status of the surgeon (i.e., consultant, specialist, resident), conversion to open surgery, and the presence of stones diagnosed during IOC. Patients who underwent transcystic exploration were evaluated, including the number of stones retrieved and conversion to open surgery.

In-hospital parameters included the number of days from hospitalization to surgery, the number of days from surgery until discharge, and the total length of stay (LOS). Factors were compared in groups of patients with and without stones on IOC and with and without preoperative dilatation of the CBD.

Finally, complications have been considered.

*χ* ^2^ or Fisher’s exact tests to compare proportions and nonparametric Mann–Whitney *U* test for analysis of values with a non normal distribution were used for statistical analysis.

## Results

Although 268 patients underwent LC for ABP during the study period, complete data were available for only 113 patients. These patients included 82 males (72.6%) and 31 females (27.4%), of mean age 46.9 **±** 14.7 years (range 18–86 years). Of these 113 patients, 14 (12.4%) were native Qataris and 99 (87.6%) were natives of other countries.

One hundred twelve patients (99.1%) presented abdominal pain, 19 patients (16.8%) jaundice, 11 patients (9.7%) fever, and 5 patients (4.4%) chills the day before admission.

During clinical examination, 9 patients (8.0%) present fever, 33 patients (29.2%) jaundice, and 90 patients (79.6%) abdominal pain.

These patients had a median total bilirubin concentration of 40.5 (18.9–71.2) mmol/L, a median ALP concentration of 153 (102.0–223.0), a median AST concentration of 174 (82.0–287.0) U/L, a median ALT concentration of 265 (137.3–387.0) U/L, a median amylase concentration of 1095.0 (375.0–2231.0) U/L, and a median lipase concentration of 3109.0 (905.5–47.38.0) U/L. Comparisons of these laboratory tests in patients with and without stones in CBD diagnosed by IOC showed that all median concentrations were higher in patients with those without stones in CBD (Table 1). But no significant differences (*P* > 0.001) between sex, nationality, abdominal pain, fever, jaundice, and chills reported by the patients or identified with clinical examinations were recorded in these two groups.

**Table 1:** Preoperative serum markers in patients with and without CBD stones intraoperatively diagnosed by IOC

ICO	Without stones CBD (29 patients)				With stones CBD (84 patients)				
	Normal value	Increased value	Median	Percentiles (25°–75°)	Normal value	Increased value	Median	Percentiles (25°–75°)	*P*
Total bilirubin 3.4–20.5 mmol/L	12	17	30.6	13.3–56.5	16	68	42.9	24.1–129.5	*P* > 0.001
ALP 40–150 U/L	9	20	158	104.5–223.0	31	53	152.5	101.5–229.3	*P* > 0.001
AST 5–34 U/L	1	28	157	62.0–280.0	6	78	181.5	93.3–291.5	*P* > 0.001
ALT 0–55 U/L	4	25	251	80.5–334.0	8	76	273.5	155–400.5	*P* > 0.001
Amylase 30–100 U/L	1	28	867	265.5–2173.0	4	80	1261.0	479.5–2397.3	*P* > 0.001
Lipase 23–300 U/L	4	25	2832	1365.5–4778.5	11	73	3223.0	800.5–4769.3	*P* > 0.001

*P* > 0.001: not significative

The group of the patients with stones in the CBD diagnosed by IOC was divided in patients with diameters <0.8 mm and with diameters ≥0.8 mm of the CBD diagnosed preoperatively with ultrasound (US). The laboratory tests reported in Table 2 do not demonstrate difference statistically significative between these two groups.

**Table 2:** Preoperative serum markers in patients with CBD stones on IOC with and without CBD dilatation on preoperative US

ICO	Patients with stones in CBD								
Preoperative US	CBD <0.8 mm (35 patients)				CBD ≥0.8 mm (49 patients)				
	Normal value	Increased value	Median	Percentiles (25°–75°)	Normal value	Increased value	Median	Percentiles (25°–75°)	*P*
Total bilirubin 3.4–20.5 mmol/L	9	26	31.9	16.1–69.9	8	41	53.0	30.0–88.5	*P* > 0.001
ALP 40–150 U/L	20	15	139.0	101.0–193.0	20	29	173.0	104.0–280.5	*P* > 0.001
AST 5–34 U/L	2	33	162.0	62.0–263.0	3	46	187.0	103.5–368.0	*P* > 0.001
ALT 0–55 U/L	3	32	244.0	141.0–384.0	3	46	280.0	170.0–440.5	*P* > 0.001
Amylase 30–100 U/L	0	35	819.0	276.0–2647.0	3	46	1390.0	490.0–2283.5	*P* > 0.001
Lipase 23–300 U/L	4	31	1438.0	763.0–4899.0	8	41	3563.0	974.5–4211.5	*P* > 0.001

*P* > 0.001: not significative

The group of the patients without stones in the CBD diagnosed by IOC was also divided in patients with diameters <0.8 mm and with diameters ≥0.8 mm of the CBD. Also in these two groups, the statistical analysis of the laboratory tests reported in Table 3 does not demonstrate significative difference.

**Table 3:** Preoperative serum markers in patients without CBD stones on IOC with and without CBD dilatation on preoperative US

ICO	Patients without stones in CBD								
Preoperative US	CBD <0.8 mm (7 patients)				CBD ≥0.8 mm (22 patients)				
	Normal value	Increased value	Median	Percentiles (25°–75°)	Normal value	Increased value	Median	Percentiles (25°–75°)	*P*
Total bilirubin 3.4–20.5 mmol/L	1	6	58.8	31.5–118.0	10	12	23.5	12.8–41.1	*P* > 0.001
ALP 40–150 U/L	3	4	158.0	66.0–479.0	11	11	158.0	94.0–176.0	*P* > 0.001
AST 5–34 U/L	1	6	157.0	126.0–212.0	1	21	155.5	57.5–292.3	*P* > 0.001
ALT 0–55 U/L	1	6	273.0	148.0–554.0	5	17	152.5	68.5–319.5	*P* > 0.001
Amylase 30–100 U/L	0	7	1013.0	572.0–2229.0	1	21	662.5	232.8–2146.0	*P* > 0.001
Lipase 23–300 U/L	0	7	3049.0	1514.0–4909.0	3	19	2427.0	1072.8–4819.6	*P* > 0.001

*P* > 0.001: not significative

Surgical procedures were performed in 33 patients (29.2%) by consultants, in 73 (64.6%) by specialists, and in 7 (6.2%) by residents. None of these 113 patients required conversion to open surgery. IOC showed stones in 84/113 patients (74.3%).

Nine patients (10.7%) on 84 with CBD stones diagnosed by IOC underwent transcystic exploration. Eight patients have their stones retrieved, using Dormia basket with multiple attempts. The clearance of the CBD has been checked using IOC. Other patients were referred to a gastroenterologist for postoperative ERCP. All explorations of the CBD were performed by consultants.

The mean time from hospitalization to surgery was 5.9 days, the mean time from surgery until hospital discharge was 2.1 days, and the mean LOS was 8.0 days. A comparison of patients with and without stones at IOC showed similar mean times from hospitalization to surgery (5.9 days [range 2–12 days] vs. 6.1 days [range 2–23 days]), from surgery until hospital discharge (2.0 days [range 0–4 days] vs. 2.2 days [range 0–11 days]), and overall length of stay (7.9 days [range 3–19 days] vs. 8.3 days [range 3–23 days]) (Table 4). Similar results were observed in patients with and without preoperative dilatation of the CBD. No statistically significant differences were identified in any group (*P* > 0.001)

**Table 4:** Times from hospital admission to surgery, from surgery to discharge, and from admission to discharge

ICO	Patients without stones in CBD (29 patients)			Patients with stones in CBD (84 patients)		
US	CBD <0.8 mm (7 patients)	CBD ≥0.8 mm (22 patients)	*P*	CBD <0.8 mm (35 patients)	CBD ≥0.8 mm (49 patients)	*P*
Days of length of hospital stay	7.1 (range 5–9 days)	9.1 (range 5–9 days)	*P* > 0.001	7.2 (range 5–9 days)	9.8 (range 5–9 days)	*P* > 0.001
Days from admission to surgery	5.4 (range 5–9 days)	6.7 (range 5–9 days)	*P* > 0.001	5.5 (range 5–9 days)	6.7 (range 5–9 days)	*P* > 0.001
Days after surgery	1.8 (range 5–9 days)	2.5 (range 5–9 days)	*P* > 0.001	1.7 (range 5–9 days)	3.1 (range 5–9 days)	*P* > 0.001

*P* > 0.001: not significative

No complications have been recorded.

## Discussion

Gallbladder stones were first correlated with ABP in 1901 [6], with this correlation later confirmed [7]. Between 3 and 7% of patients with gallbladder stones develop pancreatitis [7], especially due to passage of a small calculus (<5 mm). According to the “migratory stone” theory, 63–75% of patients with ABP develop CBD stones within 48 h of admission [7]. Similarly, we observed that 74.3% of patients retained stones at surgery. The mean time from admission to surgery in our study was about 5 days, due to the clinical conditions of the patients and to the long daily waiting list in our hospital. Following the acute episode of pancreatitis, however, only 5% of patients retained stones in the CBD [4, 7].

Stones in the CBD have been observed in patients after ABP and in patients who have never experienced ABP. The latter represents about 4.6% of all patients who undergo LC; in one-third of these patients, stones spontaneously pass 6 weeks after cholecystectomy [8]. However, a small percentage of patients who have retained their stones in the CBD may develop severe complications.

The mean age of our patients was 46 **±** 14.7 years, within the range of 37–58 years reported in earlier studies of patients undergoing cholecystectomy for ABP [9]. Although this condition has been reported to occur more frequently in women [7], we observed a male predominance, perhaps due to the admission to our country of many male workers.

The management of ABP has changed, from delayed surgery to immediate cholecystectomy. Following the resolution of pancreatitis to prevent its recurrence or other related complications, including choledocholithiasis and cholangitis, early removal of the gallbladder has been recommended [10]. Patients affected by ABP are at a 30-fold higher risk of a second attack of pancreatitis than the normal population [11]. Surprisingly, however, a longer preoperative interval has been associated with both an increased risk of ABP recurrence and a reduced risk of finding retained stones in the CBD. The likelihood of finding stones in the CBD was found to be 70% at admission, decreasing to 20% after 4 days [12]. Similarly, only 25.6% of our patients presented with stones at the time of surgery. As spontaneous stone migration in most patients is affected by pancreatitis, assessment of the CBD may be unnecessary, with ERCP performed in patients with biliary obstruction or cholangitis and total bilirubin >5 mg/ml not resolving spontaneously [11].

Early LC after ABP is defined as being performed within 1 week of admission and is currently considered the standard of treatment [8, 9]. Many patients discharged after the first episode of ABP who later undergo surgery can experience gallstone-related events (including recurrent ABP), have a prolonged length of stay, and may have adverse postoperative outcomes [13, 14].

Most of our patients underwent LC within 1 week after an episode of ABP, but some did not undergo surgery until 3 weeks after admission. Although 1 week is considered optimal, none of our patients who underwent LC after 3 weeks experienced recurrence, suggesting that the timing of the surgical procedure can be extended. Future studies are needed to determine the maximum length of this extension period.

The efficacy of IOC during cholecystectomy in patients previously affected by ABP is unclear. An intraoperative cholangiogram is usually recommended during LC to detect any retained stones that can be extracted by CBD exploration or by ERCP [9]. However, the usefulness of this procedure has not been determined.

IOC, first described in 1931 [15], is based on cannulation of the cystic duct to visualize the bile duct and has allowed the identification of bile duct stones. IOC can also enable the visualization of anatomic abnormalities, as well as the recognition of very early injuries to the CBD that can occur during surgery. Although the use of IOC is based on surgeon experience and overall preferences, no standardized criteria exist to date [16].

The time required for routine IOC has been reported to range from 8 to 20 min [17] and to depend on the availability of local resources. Other factors associated with the efficacy of routine IOC include the expertise of the specialist who analyzes the data, the exposure to radiation of persons in the operating room, and the cost of the procedure, which can vary from 100 to 700 US dollars [18]. Stone extraction can increase costs, especially if stones are expected to migrate spontaneously.

The success rate of stone extraction has been reported to range from 86–94% [19]. Failures have been reported to result from technical difficulties, especially cannulation of the cystic duct. The sensitivity and specificity of this procedure range between 93%–99%. False-positive results due to air bubbles are inevitable and can affect up to 35% of patients [20].

Postoperative stay has been reported longer in patients who did than did not undergo IOC, although the percentages of patients with retained stones in the CBD following surgery were similar (5.1 vs. 2.8%) [21]. A systematic review of six studies that included 1715 patients at low risk for choledocholithiasis randomized to undergo or not undergo IOC and followed up for 1–8 years found retained stones in only five patients, with no difference in outcomes between the two groups

All patients in our study underwent IOC, but hospitalization times did not differ in the groups of patients with and without stones. Eight patients with stones at IOC underwent stone retrieval during the laparoscopic procedure, but their postoperative LOS was similar to that in patients with stones who were not treated laparoscopically and patients without stones. A small difference in LOS was observed in patients with and without preoperatively dilated CBD (both with and without stones). The advantages of the patients submitted to the IOC, in which the stones have been diagnosed and retrieved, was to completely cure the disease at this step. Furthermore, the procedure does not have complications in relation to the group without IOC. Last, the procedure, when performed, helps young surgeons to familiarize with this intraoperative technique.

To avoid unnecessary IOC, many studies have attempted to identify preoperative factors associated with the need for IOC. Elevated bilirubin, ALP, and ALT concentrations, as well as bile duct dilatation, are considered indications for IOC in patients undergoing LC for ABP [22]. Transient increases in bilirubin, ALP, AST, and ALT concentrations can indicate papillary obstruction, due to the passage of sludge or gallstone or papillary edema. If these indicators fluctuate over a prolonged period, other methods are needed to determine the optimum time for cholecystectomy [23]. Routine intra-operative cholangiogram at the time of cholecystectomy for ABP may be unnecessary, especially if preoperative biochemical and imaging markers do not indicate an increased likelihood of CBD stones.

GGT and ALP concentrations are regarded as sensitive markers for the presence of stones in the CBD. Another study, however, reported that increases in bilirubin and ALP, as well as CBD dilatation, are not associated with abnormal cholangiograms [24]. We found that preoperative bilirubin, ALP, and GGT concentrations were similar in patients with and without stones on IOC.

A study of all patients who underwent cholecystectomy in Uppsala, Sweden, found that elevated ALP and bilirubin concentrations were the best predictors of CBD stone, but IOC yielded false positive (5%) and false negative (1%) results. Normal bilirubin and ALP levels in patients with CBD stones can be due to partial obstruction or migration of the stone before the surgical procedure. In contrast, elevated bilirubin and ALP levels in patients without CBD stones can be due to a secretory hepatic dysfunction, to a migration of stones in the duodenum, and to sludge or to CBD compression caused by other conditions [25].

Diagnosis of remnant stones in the CBD after ABP remains a challenge. Efforts to selectively manage patients with ABP, based on variables, including ultrasound findings and ALP, total bilirubin, and direct bilirubin concentrations, have attempted to identify patients requiring IOC [14]. Stratification was not possible, and the decision to perform IOC was based on the results of magnetic resonance cholangiopancreatography (MRCP). Endoscopic ultrasound can be considered to perform diagnosis of CBD stones, but is not available in all hospitals, and the results of MRCP can be comparable and more safe [26]. Another study reported that some patients positive on IOC were negative on postoperative ERCP, suggesting that IOC is unnecessary [24]. Moreover, only 2–3% of patients with markers indicative of CBD stones who did not undergo IOC were later admitted for CBD stones, again suggesting that IOC is unnecessary [8]. A recent study specifically assessing the impact of IOC on recurrent pancreatitis found that IOC did not improve the outcome after cholecystectomy for gallstone pancreatitis [10].

The limitations of the present work are the small number of the patients analyzed and that is a retrospective study.

## Conclusions

IOC is useful to diagnose residual CBD stones, without increasing complications related to the procedure itself. Although not mandatory, IOC may be useful in patients who undergo LC after ABP. Future studies are required to well elucidate this problem.

## Abbreviations

ALP: Alkaline phosphatase
ALT: Alanine aminotransferase
AST: Aspartate aminotransferase
ABP: Acute biliary pancreatitis
CBD: Common bile duct
ERCP: Endoscopic retrograde cholangiopancreatography
GGT: Gamma glutamyltransferase
IOC: Intraoperative cholangiography
LC: laparoscopic cholecystectomy
LOS: Length of stay
MRCP: Magnetic resonance cholangiopancreatography
